# Harnessing Invariant NKT Cells to Improve Influenza Vaccines: A Pig Perspective

**DOI:** 10.3390/ijms19010068

**Published:** 2017-12-27

**Authors:** Guan Yang, Jürgen A. Richt, John P. Driver

**Affiliations:** 1 Department of Animal Sciences, University of Florida, Gainesville, FL 32611, USA; guan.yang@ufl.edu; 2 College of Veterinary Medicine, Kansas State University, Manhattan, KS 66506, USA; jricht@vet.k-state.edu; 3 Diagnostic Medicine/Pathobiology and Center of Excellence for Emerging and Zoonotic Animal Diseases (CEEZAD), Manhattan, KS 66502, USA

**Keywords:** iNKT cells, CD1d, influenza vaccine, adjuvants, pig

## Abstract

Invariant natural killer T (iNKT) cells are an “innate-like” T cell lineage that recognize glycolipid rather than peptide antigens by their semi-invariant T cell receptors. Because iNKT cells can stimulate an extensive array of immune responses, there is considerable interest in targeting these cells to enhance human vaccines against a wide range of microbial pathogens. However, long overlooked is the potential to harness iNKT cell antigens as vaccine adjuvants for domestic animal species that express the iNKT cell–CD1d system. In this review, we discuss the prospect of targeting porcine iNKT cells as a strategy to enhance the efficiency of swine influenza vaccines. In addition, we compare the phenotype and tissue distribution of porcine iNKT cells. Finally, we discuss the challenges that must be overcome before iNKT cell agonists can be contemplated for veterinary use in livestock.

## 1. Introduction

Vaccines have achieved remarkable reductions in infection and disease for both humans and livestock. Indeed, global immunization regimes have successfully eradicated smallpox in humans and rinderpest in cattle, and vaccinations continue to play a key role in controlling key infections of veterinary and public importance [1]. Most vaccines produced to date have been successful for eliciting antibodies that are effective for neutralizing extracellular pathogens. However, it has been much more challenging to produce vaccines that generate sufficient numbers of long-lived cytotoxic CD8 T cells that are critical for eliminating intracellular pathogens [2]. This is partly because current vaccine formulations have difficulty in driving the kind of CD4^+^ T cell help that generates efficient cross-presentation of protective antigens for CD8 T cells [3]. Consequently, there is great interest in developing strategies that enhance vaccine-induced cellular immunity in ways that promote durable protection from disease. One approach with considerable promise is to harness the immune functions of a rare population of T cells called natural killer T (NKT) cells to enhance the immunogenicity and long-term efficacy of current vaccines.

NKT cells share phenotypic characteristics of both NK cells and T cells and are expressed by some, but not all, mammals [4]. They belong to a family of ‘innate-like’ T lymphocytes that express T cell receptors (TCRs) capable of recognizing nonpeptide antigens presented by non-major histocompatibility complex (MHC) molecules. Other family members include γδ T cells and mucosal associated invariant T (MAIT) cells. These types of lymphocytes use a narrow range of TCRs to recognize a limited repertoire of nonpeptide ligands presented by non-MHC molecules. Consequently, it is possible to target whole populations of innate-like T cells using defined antigens.

NKT cells are classified into type I and type II subsets, both of which recognize endogenous and foreign glycolipid antigens presented by the MHC class I-like CD1d molecule [5]. The type I subset, which is the focus of this review, expresses a semi-invariant TCR that recognizes the prototypic antigen α-galactosylceramide (α-GalCer) that was originally derived from the marine sponge *Agelas mauritianus* [6]. As a result, type I NKT cells, which are referred to as invariant NKT (iNKT) cells, can be detected using α-GalCer/CD1d tetramers or multimers [7]. Although type II NKT cells also bind CD1d, they express a more diverse TCR repertoire and do not recognize α-GalCer.

Often referred to as the “Swiss Army knife” of the immune system [8], activated iNKT cells provide a universal source of T cell help by rapidly producing large quantities of multiple cytokines that are capable of simultaneously activating an array of immune cell types, including NK cells [9], dendritic cells (DCs) [10], B cells [11], and conventional T cells [12]. Microorganisms have been found to activate iNKT cells directly through CD1d-bound bacterial-derived glycolipids or indirectly by the cytokines produced by antigen-presenting cells (APCs) after engagement of pattern recognition receptors (PRRs) with pathogen-associated molecular patterns (PAMPs) [13]. These responses contribute to host immunity against a variety of bacterial, viral, fungal, and protozoal pathogens [14,15,16]. In addition, iNKT cells may be therapeutically targeted with various α-GalCer derivatives in ways that stimulate and suppress immune responses. Harnessing these functions has shown potential for boosting immunity against infectious disease and tumors as well as inducing tolerance for inhibiting autoimmune disorders [17].

Since the discovery of α-GalCer, numerous studies have tested the feasibility of exploiting the adjuvant effects of this molecule and, indirectly, those of iNKT cells to improve the efficacy of vaccines (reviewed in [18]). Overall, this approach has demonstrated substantial promise, but most experiments have been carried out using mice as a model. We postulate that there exists potential to harness iNKT cells in livestock species that express iNKT cells, such as swine. Because activated iNKT cells provide a universal form of T cell help that, in many ways, is superior to currently approved adjuvants, there may be untapped potential to exploit iNKT cells, for example, to help pork producers control swine influenza infections. Apart from veterinary applications, studying iNKT cell functions in large animals like pigs offers an excellent opportunity to assess the feasibility of iNKT cell agonists for human use. Indeed, swine express similar iNKT cell subsets and frequencies compared to humans [19]. Furthermore, adaptive and innate immune cell subsets are highly homologous between these two species [20,21], which likely accounts for the susceptibility of pigs and humans to similar pathogens, including to the same influenza subtypes. Because of their similar size, pigs present a good model to better define nontoxic dosage ranges of iNKT cell therapeutics for humans [22,23]. In addition, young piglets offer the opportunity to determine whether iNKT cell therapy could be safely administered to human infants that are similarly vulnerable to influenza infections due to an immature immune system. In this review, we describe what is currently known about the iNKT–CD1d system in swine. We also summarize how iNKT cell agonists have been used to improve the efficacy and durability of influenza vaccines in mice as well as in pigs. Finally, we consider the obstacles that must be overcome before iNKT cell agonist therapy can be used for swine.

## 2. Challenges Facing the Development of Effective Swine Influenza Vaccines

Influenza A viruses (IAV) are a major cause of respiratory disease in pigs and predisposes infected animals to a host of secondary respiratory infections. Swine also act as reservoirs and intermediate hosts for influenza viruses from different animal species; these viruses sometimes undergo reassortment to produce novel strains that sporadically give rise to zoonotic infections [24], some of which are even capable of causing human pandemics. In April of 2009, a novel pandemic H1N1 virus (H1N1pdm09) of pig origin was first detected in North American human populations and quickly spread to the level of pandemic stage 6 by June 2009. The impact of this outbreak was enormous and resulted in thousands of deaths and millions of hospitalizations [25]. For the pork industry, it led to billions of dollars in lost revenue. Unfortunately, the risk of pig-derived pandemics is still relevant, due to the rapid rate at which novel swine influenza A virus (IAV-S) strains are evolving, especially since the emergence of the triple reassortant virus lineage in the late 1990s that started reassorting with additional porcine and human IAVs. This has led to much greater antigenic diversity of IAV-S strains, which has greatly complicated the development of effective vaccines.

Apart from implementing strict biosecurity, vaccines are the best defense against the spread of IAV-S infections in pigs. Yet, the coverage and efficacy of commercially available inactivated swine influenza vaccines for currently circulating IAV-S strains is often inadequate to keep up with the rapid evolution of IAV-S strains. Furthermore, intramuscularly delivered inactivated influenza virus vaccines like those used in pigs provide little cross-protection against heterologous and heterosubtypic IAV-S strains [26,27,28]. Other drawbacks of inactivated influenza vaccines include that large doses are required to induce neutralizing antibodies and that intramuscular delivery does not stimulate the high levels of secretory IgA necessary for cross-protection against heterologous viruses. Also, inactivated influenza vaccines do not elicit sufficient numbers of CD8 T cells that recognize conserved, internal components of the virus such as nucleoprotein (NP) or matrix protein 1 or 2 (M1 or M2) and, therefore, they usually fail to induce cross-protective cellular immunity [29]. Intranasally administered live attenuated influenza virus (LAIV) vaccines have shown potential for inducing antibodies as well as NP- or M-specific CD8 T cells [29,30,31]. At least three experimental LAIV vaccines have been developed for swine and some of these produce significant cross-protection against IAV-S within the same subtype. However, they offer only modest protection for pigs challenged with a different IAV-S subtype [32,33,34]. Also, LAIV vaccines for swine are not yet commercially available. Considerable effort has been spent developing various peptide-based vaccine technologies for pigs, including DNA vaccines, and replication-defective alphavirus-like replicon particles and adenovirus vectors [35]. Cross-presentation of peptides is generally superior to intact proteins and results in better CD8 T cell responses [3]. However, this approach is handicapped by the high levels of MHC polymorphism among outbred pigs that restricts the number of individuals capable of responding to a particular peptide, which is compounded by the very low frequency of naïve T cells specific for any single peptide.

There is significant potential for using adjuvants to overcome some of the above-described limitations, which are usually described as compounds that shape and/or enhance adaptive immunity [36]. When administered with vaccines, adjuvants can augment the scale, scope, and quality of an immune response. Adjuvants approved for veterinary use fall into two classes. The first includes immune stimulatory molecules such as toll-like receptor (TLR) ligands that induce the release of chemokines/cytokines from various innate immune cells that drive APC maturation. The second is delivery systems, such as liposomes, that improve antigen stability, release, or uptake [37,38]. Adjuvants currently licensed for use with inactivated IAV-S vaccines in the United States include Amphigen^®^, a lecithin and mineral oil blend; EMUNADE^®^, an oil-in-water with aluminium hydroxide formulation; and ImmunSTAR^®^, a water-in-oil-in-water emulsion. While conventional adjuvants enable dose-sparing and stimulate cellular immune responses, they usually do not provide long-term protection or robust cross-reactivity against heterologous and heterosubtypic IAV-S strains [26]. An alternative approach may be to use glycolipid ligands of iNKT cells as vaccine adjuvants, which, in addition to inducing innate immune responses, will activate iNKT cells that function as a type of ‘universal’ helper T cell to amplify both cellular and humoral immune responses against co-administered vaccine antigens [18,39,40].

## 3. Current Understanding of the Porcine iNKT Cell–CD1d System

Mouse and human iNKT cells have been extensively studied, which has helped to dissect the porcine iNKT cell–CD1d system. Both murine and human iNKT cells express a semi-invariant TCR (Vα14-Jα18/Vβ8.2, -7, or -2 in mouse or Vα24-Jα18/Vβ11 in humans); various surface molecules, including CD25, CD44, CD69, and CD122, which are expressed by activated or memory T cells; and a few lineage markers of NK cells. They also share extensive transcriptional programing with NK cells that is similar in extent to that shared with MHC-restricted T cells [41]. Subpopulations of iNKT cells analogous to Th1, Th2, and Th17 or innate lymphoid cell (ILC)1, ILC2, and ILC3 lymphocytes have been described, and many of the same transcription factors, such as T-bet, GATA-3, and RORγt, which regulate lineage commitment in these cells, also function in mouse and human iNKT cells [16,42]. In both species, iNKT cells can be divided into CD4^+^ and CD4^−^ populations, although the relative proportions of these two subsets varies widely among humans and inbred mouse strains [43,44]. Human iNKT cells that express CD4 are capable of secreting Th2 cytokines, while both CD4^+^ and CD4^−^ iNKT cells have the potential to produce Th1 cytokines [44,45]. A significant fraction of CD4^−^ human iNKT cells express CD8α, including a small population of CD8αβ cells. CD8α^+^ iNKT cells produce more interferon gamma (IFNγ) and are more cytotoxic than other iNKT cell subsets. Murine iNKT cells do not express CD8 while CD4 does not fully distinguish Th1- and Th2-producing iNKT cell subsets [16]. Other distinctions between mouse and human CD1d–iNKT cell systems include that the frequency and distribution of iNKT cells is different in most inbred mouse strains compared with humans; and that murine iNKT cells can make up 1–3% of T cells in the thymus, peripheral blood, and spleen, and up to 15% and 30% of T cells in bone marrow and liver, respectively [7,43,46,47,48,49]. The prevalence of iNKT cells in humans is typically between 0.01% and 0.1% in peripheral blood and they do not appear to be enriched in human liver [50].

Current understanding about porcine iNKT cells is rudimentary. Nevertheless, what is known suggests that they are developmentally and functionally similar to mouse and human iNKT cells. For instance, we have shown that porcine iNKT cells are CD1d restricted as CD1d knockout pigs fail to develop iNKT cells [51]. Like humans, pigs express a full complement of CD1 molecules that includes CD1a, CD1b, CD1c, CD1d, and CD1e [52] and some of these molecules can present lipid antigens [53]. In contrast, mice express two copies of CD1d, but lack the other *CD1* genes [54]. Porcine CD1d is highly homologous to mouse and human CD1d to the extent that fluorescently labeled mouse or human tetramers loaded with α-GalCer analogs can be used with equal efficiency to visualize pig iNKT cells by flow cytometry [19,51,55,56,57,58,59]. We have used this tool to show that the prevalence and distribution of iNKT cells among outbred pigs is more like humans than like mice, with the frequency of iNKT cells in blood, spleen, lung, liver, bronchoalveolar lavage fluid (BALF), tracheobronchial lymph node (TBLN), cervical lymph nodes (CLN), and mesenteric lymph nodes (MLN) typically between 0.01% and 1% of total lymphocytes [19,60]. Pig iNKT cells also express high levels of the transcription factor promyelocytic leukaemia zinc finger protein (PLZF) that is essential for the development and immunoregulatory functions of iNKT cells in other species [55]. Additional similarities include that α-GalCer causes pig iNKT cells to proliferate in vivo and in vitro, and, once activated, porcine iNKT cells can induce the downstream maturation of monocyte-derived APCs [59]. Pig iNKT cells are also capable of adjuvanting immune responses when iNKT cell agonists are co-administered with antigens, with the effect of generating antigen-specific humoral and cellular responses that are useful for augmenting vaccines [19].

Some ways that porcine iNKT cells differ from mouse and human iNKT cells include their unique pattern of CD4 and CD8α co-receptor expression; and their lack of a distinct CD4-expressing population, although a small number of iNKT cells display low levels of CD4, especially in the lymph nodes and spleen [59]. Most porcine iNKT cells are CD8α positive [59] (and CD8β negative (Driver et al., University of Florida, Gainesville, FL. Characterizing porcine NKT cell subsets, 2017)), which is not surprising as swine are known for their high proportion of lymphocyte subsets that express the CD8α co-receptor, which mostly form homodimers on the cell surface [61,62]. In pigs, expression of CD8α on CD4^+^ T cells has been associated with the acquisition of memory function as antigen-specific recall immune responses of memory T-helper cells are within the CD4^+^ CD8α^+^ T cell population [63,64,65,66]. Likewise, CD8α expression may be linked to the acquisition of memory function by porcine iNKT cells as, in other species, these cells constitutively express surface markers that are characteristic of memory T cells. In some tissues, porcine iNKT cells can be separated into CD8α bright and dull populations that respectively produce higher and lower levels of IFNγ upon stimulation [59]. It is possible that the CD8α expression level may distinguish subtypes of iNKT cells or iNKT cells in higher or lower states of activation.

At the time of writing, information about subsets of porcine iNKT cells is minimal. Our efforts to differentiate populations according to surface molecules, cytokines, and transcription factors that distinguish iNKT cell subsets in other species have been hampered by the limited availability of reliable reagents available for pigs. We have shown that porcine iNKT cells do not express CD11b, although this molecule is expressed by ~10% of mouse iNKT cells; this value expands to >90% after bacterial infection [67]. In contrast, all pig iNKT cells express CD44, which is a molecule acquired by mouse and human iNKT cells as they undergo maturation in the thymus [68,69,70]. However, CD44 is expressed at the same high level on iNKT cells as on conventional T cells, which is consistent with reports that CD44 is expressed by most swine lymphocyte populations [71]. Pig iNKT cells from the blood and liver rapidly synthesize IFNγ after activation with phorbol myristate acetate (PMA)/ionomycin, which is consistent with other species. However, interleukin (IL)-4 is difficult to detect. We are also unable to identify IL-17^+^ iNKT cells even though stimulation with PMA/ionomycin is sufficient to induce substantial IL-17 production from murine iNKT cells [72]. In this way, porcine iNKT cells may be more like human iNKT cells that require a combination of transforming growth factor beta (TGF-β), IL-1β, and IL-23 to induce the production of IL-17 [73].

## 4. Universal Helper T Cell Functions of iNKT Cells

**Figure 1:**
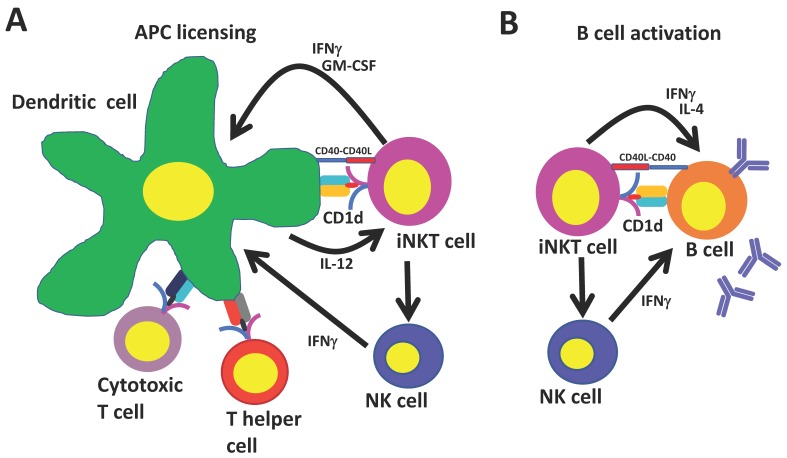
Universal T helper cell functions of invariant natural killer T (iNKT) cells. (**A**) iNKT cells mature and license dendritic cells when they recognize agonist-bound CD1d. iNKT cells upregulate CD40L and secrete interferon gamma (IFNγ) and granulocyte-macrophage colony-stimulating factor (GM-CSF), which induces costimulatory molecule expression and the production of interleukin (IL)-12 by dendritic cells. This further stimulates iNKT cells and primes CD8^+^ T cells against copresented peptide epitopes. iNKT-conditioned dendritic cells promote conventional CD4^+^ T cell help to peptide antigens, which leads to additional enhancement of CD8^+^ T cell responses and promotes the generation of follicular T helper cells; (**B**) iNKT cells binding cognate antigen on CD1d-expressing B cells differentiate into iNKT follicular helper cells, which resemble conventional helper cells in their ability to augment B cell responses through a CD40–CD40L dependent mechanism. iNKT cells may also boost humoral immunity by transactivating NK cells that can stimulate B cells to secrete immunoglobulin G (IgG).

Because the CD1d molecule is non-polymorphic, iNKT cells can be specifically and globally activated with glycolipid ligands. Immune responses induced by activated iNKT cells resemble conventional CD4^+^ T cell help in several respects (Figure 1): iNKT cells mature and license dendritic cells when they recognize agonist-bound CD1d on their surface. Recruited iNKT cells in turn become activated and upregulate CD40L and secrete IFNγ and granulocyte-macrophage colony-stimulating factor (GM-CSF) which induces costimulatory molecule expression and the production of IL-12 by dendritic cells [74,75]. This stimulates further iNKT cell activation and primes CD8^+^ T cells against copresented peptide epitopes. In addition, iNKT-conditioned dendritic cells promote conventional CD4^+^ T cell help to peptide antigens, which leads to additional enhancement of CD8^+^ T cell responses and promotes the generation of follicular T helper cells that are important for generating humoral responses. iNKT cells may also boost humoral immunity by transactivating NK cells that can stimulate B cells to secrete immunoglobulin G (IgG) [76].

Studies in MHCII knockout mice that express iNKT cells but lack conventional CD4^+^ T cells have shown that iNKT cells can generate humoral immunity independently of their effects on conventional CD4^+^ T cells [77]. Co-administration of iNKT cell ligands with protein antigens to MHCII knockout mice stimulates germinal center formation, followed by class switching and affinity maturation of antigen-specific B cells and the generation of long-lasting B cell memory. These effects depend on iNKT cells binding their cognate antigen on CD1d-expressing B cells which triggers iNKT cells to differentiate into iNKT follicular helper cells, which resemble conventional helper cells in their ability to augment B cell responses through a CD40–CD40L dependent mechanism [11,78,79,80]. Furthermore, during viral infections, interfollicular iNKT cells may affect B cell responses by secreting a wave of IL-4 that triggers the seeding of germinal center cells and by activating antigen-experienced B cells [81].

The advantage of eliciting T helper cell responses from iNKT cells rather than conventional CD4^+^ T cells is the relative abundance of iNKT cells compared to antigen-specific CD4^+^ T cells, which make up a minute fraction of the CD4^+^ T cell compartment. While iNKT cells constitute between 0.01% and 0.1% of total T cells in humans and up to 1% of T cells in pigs, fewer than 0.005% of CD4^+^ T cells may be specific for any single antigen [82]. Conventional helper T cell responses are also restricted by the high level of MHC polymorphism that exists among individuals in an outbred population. Co-administration of glycolipid ligands that specifically and simultaneously target large numbers of iNKT cells at the time of vaccination has unique potential for generating an extensive and multifaceted immune response of the kind that stimulates durable protection against infection.

## 5. Adjuvanting Influenza Vaccines with iNKT Cell Agonists—A Brief History 

α-GalCer analogs have been tested as adjuvants for a variety of viral vaccines [83,84]. However, most research in this area has focused on mouse models of influenza as the systems are well established, mice are easy to work with, and compatible with a wide range of vaccine technologies. So far, at least 10 studies have been published using a variety of vaccine formats, delivery routes, and mouse strains (Table 1). All have shown that iNKT cell activation significantly improves the efficacy of IAV vaccine formulations. Furthermore, iNKT cell responses appear to perform as well or better than currently approved adjuvants for stimulating long-term protection and cross-reactivity to heterologous IAV strains, which is of great practical importance. To our knowledge, Ko et al. were the first group to explore adjuvanting influenza vaccines with iNKT cell agonists [85]. They demonstrated how BALB/c mice, which were administered α-GalCer and A/PR8/8/34 hemagglutinin (PR8 HA) derived from H1N1 influenza strain A/PR8/8/34 via the intranasal route, were much better protected from homologous virus infection than mice immunized with the PR8 HA alone. α-GalCer-mediated disease resistance was associated with high levels of mucosal IgA and systemic IgG antibodies. Mice in this study were challenged two weeks after they received three vaccinations one week apart. However, the same group later published that a single intranasal vaccination with formalin-inactivated PR8 virus and α-GalCer was sufficient to elicit long-lasting IgA and IgG responses that provided durable protection when mice were challenged three months after vaccination [86].

Kamijuku et al. assessed whether a similar vaccination strategy could be used to generate cross-protection against heterologous and heterosubtypic IAV strains [87]. BALB/c mice were immunized with A/PR8 (H1N1), A/Yamagata (H1N1), A/Guizhou (H3N2), or B/Ibaraki HA vaccines with α-GalCer twice with a four week interval and intranasally (i.n.) challenged with various influenza viruses. Strong protection was generated against heterologous IAV strains within the same HA vaccine subtype and modest protection against heterosubtypic strains. The contribution of α-GalCer to protection was unclear as HA vaccination alone was not tested. However, in a subsequent experiment, the same authors showed that an inactivated whole-virion vaccine of H5N1 influenza elicited substantial cross-protection against a heterologous H5N1 IAV strain when administered with, but not without, α-GalCer. A similar regimen using cytokine knockout mice revealed that the adjuvant effects of α-GalCer stimulate mucosal IgA production and improve survival from infection through immune responses that require IL-4 but not IFNγ [87]. To visualize the dispersal of mucosal delivered α-GalCer, the authors treated mice i.n. with Cy3-labeled α-GalCer and analyzed for the distribution of α-GalCer-containing cells. Most α-GalCer remained localized to the nasal-associated lymphoid tissue (NALT) and CLN where it was stored within the intracellular vesicles of DCs. α-GalCer-containing DCs co-localized with iNKT cells that accumulated at these sites through a process involving the chemokine receptor CXCR6 and its ligand CXCL16. Only a small number of Cy3-positive cells transiently reached the spleen while even fewer migrated to the liver.

Other reports that used α-GalCer as an intranasal vaccine adjuvant include a study where the α-GalCer derivative α-C-galactosylceramide (α-C-GalCer) enhanced immune responses from an attenuated PR8 influenza virus encoding an NS1 protein truncation [88]. NS1 is needed to inhibit key components of the host innate immune system [89,90,91,92,93]. BALB/c mice were challenged with wildtype virus 21 days after vaccination with NS1-truncated attenuated virus and α-C-GalCer at a range of doses (0–4 μg/mouse). Survival of mice vaccinated with the attenuated virus and 1 μg of α-C-GalCer was 80%, while none of the mice that received the virus and 4 μg or vaccine alone survived. This outcome should raise a cautionary note about ‘over-activating’ iNKT cells as this could inhibit replication of LAIV after vaccination, probably through heightening innate responses. Another study tested whether two glycolipid derivatives—KB-007 and KB-009—with branched acyl chains that afford improved solubility compared with α-GalCer could be used to adjuvant a formalin-inactivated PR8 vaccine [94]. KBC-009 enhanced cytotoxic T lymphocyte (CTL) responses to a greater extent than α-GalCer. However, neither analog performed better than α-GalCer for improving survival or weight recovery. Recently, investigators tested α-GalCer–peptide conjugate vaccines comprising synthetic long-peptides (SLP) from an IAV-specific protein covalently attached to α-GalCer via a cleavable linker [95]. Conjugating peptides with α-GalCer ensures that vaccine and adjuvant are delivered to the same APC, which enables activated iNKT cells to license DCs involved in stimulating CTL responses against the peptide antigen. Other advantages of SLP are that they are highly defined and easy to manufacture and that synthesizing SLP from the target protein covering the entire sequence ensures CD4 and CD8 T cell epitopes are present for individuals with different HLA haplotypes. As proof-of-principle, C57BL/6 mice were immunized with SLP-conjugated vaccines that were synthesized based on an immunogenic peptide from chicken ovalbumin (OVA_57_). Mice were challenged 6–8 weeks later with a recombinant IAV expressing OVA. Animals that received the OVA-recombinant vaccine were completely protected, while those immunized with the backbone virus were not. Whether this approach provides protection against non-recombinant IAVs remains to be determined.

Most reports describing iNKT cell agonists as IAV vaccine adjuvants immunize via the intranasal route because the nasal mucosa is important for host defense against air-borne pathogens. However, α-GalCer-adjuvanted vaccines have also been shown to induce effective IAV immunity when delivered by alternative methods. Kamijuku et al. found that mice immunized with α-GalCer and various HA subunit vaccines via intramuscular injection were strongly protected from homologous virus infection but not from a heterologous challenge [87]. Galli et al. demonstrated that mice immunized intraperitoneal (i.p.), subcutaneous (s.c.), intramuscular (i.m.), or intraveneous (i.v.) with α-GalCer and various proteins, including HA/NA subunits from IAVs, developed antibody titers 1–2 logs higher than mice immunized with protein alone [78]. The results were similar among BALB/c, CD1, and C3H/HeJ mice, indicating that the adjuvant effects of iNKT cells are largely independent of strain and route of administration. Interestingly, consistent antibody responses were not achieved after i.n. administration of proteins with α-GalCer, which contradicts other reports that used this route. It was also observed that α-GalCer is at least equivalent to standard adjuvants (Complete Freund’s Adjuvant (CFA), CpG, and Alum), for inducing antigen-specific antibodies, especially at low antigen doses. Furthermore, α-GalCer was similarly effective to MF59 (a squalene-in-oil emulsion licensed for use with human flu vaccines) for enhancing an H1N1 subunit vaccine against infection with a heterologous IAV.

Inducing sufficient numbers of cross-reactive CD8^+^ T cells remains a major barrier for generating immunity against serologically distinct IAV subtypes [96]. Hence, Guillonneau et al. investigated whether α-GalCer-adjuvanted IAV vaccines could be used to improve heterosubtypic CTL responses [97]. Six weeks after s.c. injection with an inactivated PR8 (H1N1) vaccine, alone or with α-GalCer, C57BL/6 mice were infected with IAV HKx31 (H3N2). iNKT cell activation paradoxically diminished the acute CTL response through iNKT cell-dependent expression of indoleamine 2,3-dioxygenase (IDO), which renders effector T cells inactive while simultaneously promoting the survival of long-lived memory CD8^+^ T cells capable of boosting virus clearance from the lungs. The induction of these memory cells appeared to be due to the upregulation of prosurvival genes, including bcl-2, and indicates that activated iNKT cells have potential for boosting vaccine-induced CTL responses capable of mediating immunity against conserved viral epitopes. In addition to protein-based vaccines, α-GalCer analogs have potential for increasing the potency of DNA vaccines against influenza. Hung et al. vaccinated BALB/c mice i.m. with different α-GalCer derivatives in combination with DNA vaccines generated using a consensus HA based on available H5N1 sequences where the most conserved amino acid at each position was chosen to create the consensus HA [98]. iNKT cell activation greatly increased the capacity of H5 DNA vaccines to induce cellular and humoral immune responses and resistance against virus challenge, while also increasing their cross-neutralization capacity against a broad spectrum of H5N1 virus clades. A recent study using α-GalCer as an adjuvant of a DNA vaccine encoding influenza A virus M2 found higher IgG titer and IFNγ and IL-4 production in DNA-vaccinated mice [99]. These responses were associated with improved survival rate and reduced body weight loss after live virus challenge [99]. Thus, iNKT cell adjuvants may help overcome an important limitation of DNA vaccines—that they elicit only modest immunity in humans, which has hampered attempts to gain FDA approval for this technology.

While mouse studies provide strong support for using α-GalCer analogs to improve IAV vaccines, iNKT cell activation failed to improve IAV immunity when attempted in macaques, which represent a valuable nonhuman primate model of influenza. Indeed, no clear increase in IAV-specific cellular or humoral immune responses was detected following intravenous co-administration of α-GalCer with a LAIV compared with the LAIV alone [100]. This may be related to the method of vaccine administration rather than the animal model as α-GalCer was incubated with whole blood for two hours before delivery at the same time as the first inoculation of LAIV. Due to differences in pharmacokinetics between the vaccine constituents, this approach may limit the uptake of adjuvant and antigen by the same APC that is essential for iNKT cell help to fully stimulate influenza-specific T or B cell immunity.

## 6. Use of iNKT Cell Agonists as Adjuvants in Swine

We first established the adjuvant potential of iNKT cell agonists for swine by injecting commercial outbred pigs with the model antigen hen egg lysozyme (HEL), alone or in combination with one of the three glycosphingolipids—α-GalCer, C-glycoside, and OCH—that differentially activate iNKT cells in mice and humans [19]. While α-GalCer has exceptionally high stimulatory capacity and induces both Th1 and Th2 cytokines, C-glycoside and OCH respectively promote Th1 and Th2 immune responses [101,102,103,104]. After two immunizations, all three analogs, but particularly OCH, generated high levels of HEL-specific antibodies in pigs compared with immunization with HEL alone. The improved response from OCH may be because this agonist favors the production of Th2 cytokines that promote antibody production [101,102]. Vaccinating pigs with α-GalCer generated more HEL-specific T cells than OCH and C-glycoside, perhaps because this analog elicits a comparatively stronger and more mixed cytokine response for maturing and licensing APCs, which is required for cross-presentation of antigens to CD8^+^ T cells.

We later demonstrated that α-GalCer is an effective adjuvant for enhancing flu vaccines in swine, by vaccinating newly-weaned piglets i.m. with ultraviolet (UV)-killed whole influenza virus (pandemic H1N1 (pH1N1) A/California/04/2009 (kCA04)) alone or in combination with α-GalCer, and challenging with the live virus two weeks later [57]. α-GalCer administration strongly inhibited viral replication in the upper and lower respiratory tract and greatly reduced viral shedding; this was associated with a systemic increase in iNKT cells, including in the respiratory tract, and high levels of antihemagglutinin antibodies. Surprisingly, although there was tremendous variation in iNKT cells among pigs receiving α-GalCer, no association was found between iNKT cell concentration and antibody responses or disease protection. In contrast, virus-specific T cell responses were correlated with iNKT cell frequencies, which may be important for generating long-lasting memory and cross-protection against viral infections, but these outcomes were not tested and warrant further investigation.

To mimic commercial swine influenza vaccine application, our group employed injections into the neck muscle. However, Dwivedi et al. demonstrated that α-GalCer also potentiates influenza immunity via the intranasal route. α-GalCer co-administered with a UV-inactivated H1N1 (Sw/OH/24366/07, SwIV OH07) enhanced virus-specific IgA secretion, NK cell cytotoxicity, and Th1 cytokine (IFNγ and IL-12) secretion, and reduced immunosuppressive cytokine (IL-10 and TGF-β) production in the lungs after homologous challenge [56]. This was associated with a 3-log reduction of viral load in the lungs. Taken together, these limited number of pig studies indicate that α-GalCer analogs elicit similar adjuvant effects in pigs as they do in mice, which can boost protective immunity when applying inactivated whole-virus influenza vaccines.

## 7. Challenges to Applying iNKT Cell Agonists in Swine

Several hurdles must be overcome before iNKT cell agonists can be considered for adjuvanting swine influenza vaccines. An important obstacle is synthesizing and licensing α-GalCer analogs in a way that is financially viable. Currently, a small number of manufacturers supply α-GalCer derivatives for research purposes at a cost that would be prohibitive for veterinary use. Even if mass produced, the expense of synthesizing α-GalCer analogs will likely exceed that of currently approved adjuvants. Thus, to succeed, it will be necessary for iNKT cell agonists to deliver significant benefits over existing adjuvants, such as long-lived CTL responses and enhanced heterologous cross-protection. Another concern is whether the large range of interindividual variation in iNKT cell frequencies among outbred pigs will influence the efficacy of iNKT cell adjuvants. This may not be critical as we found the adjuvant effects of α-GalCer equally effective in pigs with diverse iNKT cell frequencies. It has been reported that iNKT cells become hyporesponsive after a second systemic administration of α-GalCer [105,106,107,108], which is important if this strategy is to be used for multiple vaccinations. However, iNKT cell hyporesponsiveness does not develop in mice vaccinated i.m. with low-doses of α-GalCer. Furthermore, porcine iNKT cells proliferate to the same extent after a first and second injection of α-GalCer spaced two weeks apart, indicating that they do not become anergized [19,57]. Another concern is whether α-GalCer analogs can be transferred to humans through meat, which must be addressed in order to gain regulatory approval. One pharmacokinetic analysis in humans showed that α-GalCer quickly disappears from the blood with no evidence for drug accumulation in serum, even with weekly administrations over a wide range of doses [105], and the same is likely to be true in swine. However, whether α-GalCer is retained in tissues remains to be determined. It will also be necessary to conduct toxicity studies to address general safety issues of iNKT cell agonists in swine, including their potential to induce local and systemic reactions. It is important that such studies evaluate α-GalCer in the context of synergy between α-GalCer-induced reactions and influenza-induced responses. Another concern is whether α-GalCer administration influences vaccine-associated enhanced respiratory disease (VAERD). This phenomenon develops from the use of inactivated vaccines containing a virus of the same hemagglutinin subtype as the subsequent challenge strain, but with substantial antigenic shift [109]. A consistent predisposing feature of VAERD is the presence of IgG antibodies that cross-react with the heterologous virus proteins but lack the ability to hemagglutinize or neutralize the heterologous virus effectively. Instead, these antibodies appear to bind immunodominant epitopes in the HA stem region (HA2) of the heterologous virus, causing enhanced virus fusion activity to the host cell that results in increased infection [109]. It is important to determine whether such reactions are worsened by adjuvants, including iNKT cell agonists, which may boost non-neutralizing antibody responses against heterologous viruses.

## 8. Concluding Remarks

Broadly protective vaccines for influenza viruses are urgently sought for humans and livestock due to their tremendous prospect to limit mortality, morbidity, and the economic burden caused by seasonal and pandemic influenza. However, traditional vaccine adjuvants do not generate optimal humoral and cellular immunity partly because of inadequate T cell help from CD4^+^ T cells. Harnessing iNKT cell functions holds considerable promise for improving vaccines, including for influenza, because they can be specifically and globally activated using glycolipid antigens to provide a universal form of T cell help that boosts peptide-specific CD8^+^ T cell and antibody responses. Data from a limited number of swine studies suggests that pigs are similar to mice in the way they respond to the adjuvant effects of α-GalCer, even though the frequency and distribution of iNKT cells between these species is quite distinct. Since this is the case, there are many potential benefits to pursuing iNKT cell therapies in pigs, including the development of better swine influenza vaccines, which could reduce the occurrence of influenza pandemics in humans. There is also the opportunity to establish the feasibility and safety of using α-GalCer analogs to improve human vaccines, as swine and humans share important similarities that make pigs an excellent preclinical model for human influenza vaccines. In summary, the well-described and long-established swine influenza challenge model offers a way to better understand the adjuvant potential of activating iNKT cells for influenza vaccines in a natural host species. This knowledge could be utilized in both humans and swine to limit the current cycle of swine-to-human transmission of influenza viruses.

## Figures and Tables

**Table 1:** Summary of murine studies using iNKT cell agonists as influenza vaccine adjuvants.

Strain	Vaccination		Details	NKT Cell Agonist (Dose per Mouse)	Ref.
	Route	Strain/Subunit			
BALB/c	i.n.	H1N1 PR8	Immunization with PR8 HA antigen with αGC 3 times at 1-week intervals, infection with 20 LD_50_ PR8 2 weeks after final immunization	αGC (0.125, 0.5, 2 µg)	[85]
BALB/c	i.n.	H1N1 PR8	Immunization with inactivated PR8 with αGC, infection with 20 LD_50_ PR8 2 weeks and 3 months after immunization	αGC (0.5 µg)	[86]
BALB/c	i.n./i.m.	H1N1 PR8 H1N1A/Yamagata H3N2A/Guizhou B/Ibaraki	Immunization with PR8, A/Yamagata, A/Guizhou, or B/Ibaraki HA vaccine with αGC twice at 4-week apart, infection with 40 LD_50_ PR8 2 weeks after second immunization	αGC (2 µg)	[87]
BALB/c	i.n.	H1N1 PR8	Immunization with PR8 with αGC twice at 4-week apart, infection with 40 LD_50_ A/Yamagata, A/Guizhou, or B/Ibaraki 2 weeks after second immunization	αGC (2 µg)	[87]
BALB/c	i.n.	H5N1 NIBRG14	Immunization with NIBRG14 inactivated vaccine with αGC twice at 4-week apart, infection with 10^3^ PFU of A/Vietnam or A/HK483 influenza virus 2 weeks after second immunization	αGC (2 µg)	[87]
BALB/c	i.n.	H1N1 rNS1 1-73	Immunization with rNS1 1-73 with different amounts of α-C-GC, infection with 100 LD_50_ PR8 3 weeks after immunization	α-C-GC (0.11, 0.33, 1, 2, 3, 4 µg)	[88]
BALB/c	i.n.	H1N1 PR8	Immunization with inactivated PR8 with αGC or αGC analogues, infection with 5 LD_50_ PR8 4 weeks after immunization or 100 LD_50_ PR8 5 weeks after immunization	αGC (0.5 µg) KBC-007 (0.5 µg) KBC-009 (0.5 µg)	[94]
C57BL/6	i.v.	H1N1 PR8-OVA257	Immunization with SLP-conjugated vaccine PR8-OVA257 with αGC, infection with 10^4^ PFU HKx31-OVA257 6–8 weeks after immunization	αGC (76 ng)	[95]
BALB/c CD1 C3H/JeJ	i.p., s.c., i.m., i.v.	HA/NA from H3N2 PNM07	Immunization with PNM07 protein with αGC for twice at 2-week apart, analysis of protein-specific antibodies	αGC (0.1 µg)	[78]
C57BL/6	i.m.	H1N1 NC20	Immunization with NC20 protein with αGC twice at 2-week apart, infection with 100 LD_50_ H1N1 A/WS/33 2 weeks after second immunization	αGC (0.1 µg)	[78]
C57BL/6	i.m.	H3N2 PNM07	Immunization with H3N2 PNM07 protein with αGC twice at 0 and 2 weeks, boosted with PNM07 at 30 weeks	αGC (0.1 µg)	[78]
C57BL/6	s.c.	H1N1 PR8	Immunization with inactivated PR8 with αGC, infection with 10^4^ PFU of live H3N2 HKx31 6 weeks after immunization	αGC (1 µg)	[97]
BALB/c	i.m.	pCHA5 for H5N1	Immunization with pCHA5 with C34 with αGC for twice at 3-week apart, infection with 200 LD_50_ NIBRG14 2 weeks after immunization	C34 (2 µg)	[98]
BALB/c	i.m.	DNA vaccine encoding M2	Immunization with DNA vaccine encoding M2 with αGC for 3 times at 2-week intervals, infection with 1 LD_90_ PR8 2 weeks after final immunization	αGC (1 µg)	[99]

PR8: H1N1 strain A/Puerto Rico/8/34; NC20: H1N1 strain A/New Caledonia/20/99; A/WS/33: H1N1 strain A/Wilson-Smith/1933; PNM07: H3N2 strain A/Panama/2007/99; A/Yamagata: H1N1 strain A/Yamagata/120/86; A/Guizhou: H3N2 strain A/Guizhou/54/89; B/Ibaraki: B/Ibaraki/2/85; rNS1 1-73: A PR8 mutant virus expressing only the first 73 amino acids in the NS1 gene; NIBRG-14: reassortant virus derived from PR8 and A/Vietnam/1194/2004 (H5N1) virus (in which the polybasic HA cleavage site has been excised); α-C-GC: α-C-galactosylceramide; αGC: α-galactosylceramide; i.m.: intramuscular; i.n.: intranasal; i.p.: intraperitoneal; s.c.: subcutaneous; i.v.: intravenous.

## Abbreviations

iNKT	Invariant natural killer T
TCR	T cell receptor
MHC	Major histocompatibility complex
MAIT	Mucosal-associated invariant T
α-GalCer	α-galactosylceramide
DC	Dendritic cell
APC	Antigen-presenting cell
PRR	Pattern recognition receptor
PAMP	Pathogen-associated molecule pattern
IAV	Influenza A viruses
IAV-S	Swine influenza A virus
NP	Nucleoprotein
M1 or M2	Matrix protein 1 or 2
LAIV	Live attenuated influenza virus
TLR	Toll-like receptor
ILC	Innate lymphoid cell
IFNγ	Interferon gamma
BALF	Bronchoalveolar lavage fluid
TBLN	Tracheobronchial lymph node
CLN	Cervical lymph nodes
MLN	Mesenteric lymph nodes
PLZF	Promyelocytic leukaemia zinc finger protein
PMA	Phorbol myristate acetate
IL	Interleukin
TGF-β	Transforming growth factor beta
GM-CSF	Granulocyte-macrophage colony-stimulating factor
IgG	Immunoglobulin G
PR8 HA	H1N1 strain A/Puerto Rico/8/34 hemagglutinin
i.n.	Intranasal
NALT	Nasal-associated lymphoid tissue
CTL	Cytotoxic T lymphocyte
SLP	Synthetic long-peptides
OVA	Ovalbumin
i.p.	Intraperitoneal
s.c.	Subcutaneous
i.m.	Intramuscular
i.v.	Intraveneous
CFA	Complete Freund’s Adjuvant
IDO	Indoleamine 2,3-dioxygenase
HEL	Hen egg lysozyme
UV	Ultraviolet
VAERD	Vaccine-associated enhanced respiratory disease
